# Cell Death and Unprecedent Cytoskeletal Changes in *Leishmania amazonensis* Parasites Evidenced by 3D Reconstruction

**DOI:** 10.1021/cbmi.6c00109

**Published:** 2026-06-25

**Authors:** Rayanne Regina Beltrame Machado, Amanda Beatriz Kawano Bakoshi, Tânia Ueda-Nakamura, Sueli de Oliveira Silva Lautenschlager, Maria Helena Sarragiotto, Danielle Lazarin-Bidóia, Celso Vataru Nakamura

**Affiliations:** † Laboratório de Inovação Tecnológica no Desenvolvimento de Fármacos e Cosméticos, Departamento de Ciências Básica da Saúde, 42487 Universidade Estadual de Maringá (UEM), Maringá, Paraná 87020-900, Brazil; ‡ Programa de Pós-graduação em Química, Departamento de Química, Universidade Estadual de Maringá (UEM), Maringá, Paraná 87020-900, Brazil

**Keywords:** Leishmania amazonensis, bithiophene, cell death, three-dimensional reconstruction, cytoskeleton

## Abstract

Leishmaniasis remains a neglected tropical disease with limited and highly toxic therapeutic options. In search of safer and more effective alternatives, bithiophene derivatives have emerged as promising scaffolds. In this study, we evaluated the antileishmanial potential of **BT-Ac**, a bithiophene previously shown to exert antitrypanosomal activity. **BT-Ac** displayed selective antiproliferative activity with IC_50_ for 28.9 and 50 μM against promastigotes and amastigotes of *Leishmania amazonensis* at nontoxic concentrations for host cells, achieving a selectivity index of 4.69 against intracellular amastigotes. Electron microscopy revealed early morphological damage in promastigotes, including unprecedented changes at the plasma membrane and cytoskeletal interface, mitochondrial swelling, lipid body accumulation, and autophagic vacuole formation. Three-dimensional reconstructions further validated these findings, confirming that the ultrastructural alterations were intrinsic to **BT-Ac** treatment rather than technical artifacts. Biochemical analyses demonstrated reactive oxygen and nitrogen species overproduction, lipid peroxidation, and ATP fluctuations, indicating oxidative stress as a central mechanism. These events culminated in apoptosis-like and autophagic death in promastigotes, while amastigote death was primarily mediated by macrophage activation, evidenced by nitric oxide release. Together, our findings identify **BT-Ac** as a selective leishmanicidal agent that disrupts parasite homeostasis through oxidative damage, highlighting its potential as a lead compound for therapeutic strategies against leishmaniasis.

## Graphical abstract

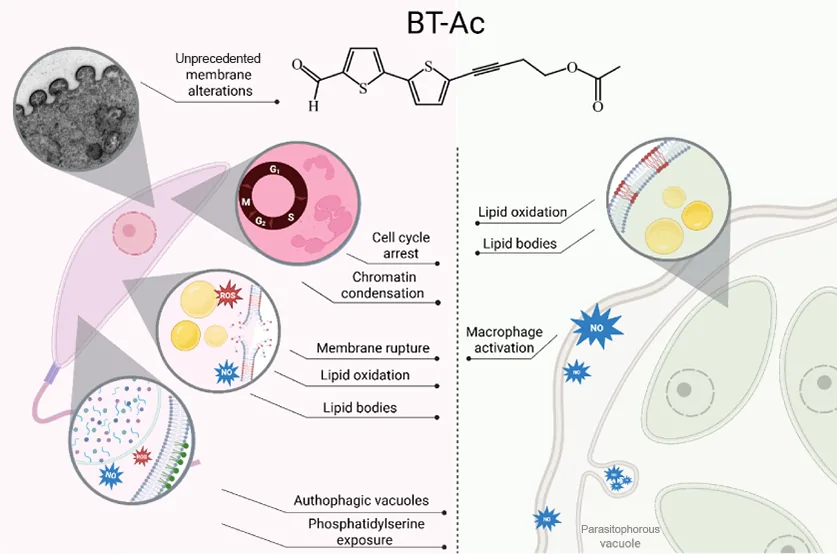

## 1 Introduction

Leishmaniasis is a group of vector-borne diseases caused by obligate intracellular protozoan parasites (Trypanosomatidae family). At least 20 species of *Leishmania* spp. are responsible for several clinical manifestations and the parasites are transmitted between mammalian hosts by sand-flies. The disease is prevalent across tropical and temperate regions, primarily found in developing areas of the world and it is categorized as a neglected tropical disease (NTD).^1,2^ It is associated with numerous risk factors, such as poverty, malnutrition, migration, and poor living conditions. Climate change, alterations in environmental conditions and human behaviors, including travel habits, an increasing number of immunosuppressed people and migration also contribute to the spread of vectors and the broader transmission of leishmaniasis.^3^

According to the World Health Organization, an estimated 10 to 15 million people worldwide are infected, with 700 thousand to 1 million new cases reported annually.^4,5^ Despite some countries reducing the number of visceral leishmaniasis cases, countries in the South Caucasus continue to have high rates of cutaneous leishmaniasis. Its prevalence is growing in areas like Syria, Turkey, and Jordan, driven by conflict-related migration and the resulting increase in refugee populations.^6,7^

The clinical manifestations of leishmaniasis are mainly determined by the *Leishmania* species involved and the host’s immune response.^8^ Mostly, these parasites exhibit a digenetic life cycle involving both a mammalian host and an insect vector, and similar to other trypanosomatid parasites, certain developmental stages are proliferative, while others are quiescent and preadapted for transmission to the next host. *Leishmania* spp. displays two main cell morphologies: the promastigote form in the sand fly with an elongated body with an externalized flagellum and high motility; these forms are responsible for infecting host cells and subsequently differentiating into amastigotes, the intracellular form, which have a typically smaller and spherical body with an internalized flagellum.^9,10^ Despite these morphological differences, the fundamental cellular architecture is conserved across both forms and is characterized by a network of cross-linked subpellicular microtubule corsets. This microtubule array is preserved throughout the cell cycle, making cell division dependent on the insertion and elongation of microtubules into the preexisting structure. The precise definition of *Leishmania* spp. cell morphology is based on parameters such as cell shape, flagellum length, and the positions of the kinetoplast and nucleus, as well as other ultrastructural features, which have been traditionally used to differentiate the observed cell forms.^11,12^

Advances in cell biology are essential not only for understanding the cell cycle and host–pathogen interactions but also for identifying novel drug targets and elucidating their mechanisms of action. In this context, 3D imaging technologies have significantly advanced and correlative microscopy techniques have enabled large-volume 3D imaging of tissues and single cells while preserving subcellular detail.^13,14^ Volume electron microscopy, particularly focused ion beam scanning electron microscopy (FIB/SEM), has further enhanced high-resolution 3D reconstruction of cellular structures, including protozoan organelles.^15–17^

No effective human vaccine is currently available against any *Leishmania* species, and treatment relies on a limited range of drugs. Chemotherapy is based on the use of pentavalent antimonials as first-line drugs (Pentostam and Glucantime), in addition to pentamidine, amphotericin B (in free or liposomal forms), paromomycin, and miltefosine as second-line drugs. Although these medications are generally effective, they present several limitations, such as host toxicity, prolonged treatment duration, widespread parasite resistance, and high cost.^18–20^ Considering the disadvantages and limitations of the available treatments, there is a pressing need to search for new drugs for the treatment of leishmaniasis. In this context, our research group has demonstrated that a class of compounds, bithiophenes, exhibits antileishmanial activity against trypanosomatids, with several molecules being active against *L. amazonensis* ^21^ and **BT-Ac** (Figure 1) showing notable efficacy against *Trypanosoma cruzi* ^22^ and immunomodulatory effect.^23^ Building on these findings, the present study investigated the leishmanicidal potential of **BT-Ac** against both promastigote and amastigote forms of *L. amazonensis*. The compound’s mechanism of action was explored through morphological, biochemical analysis and three-dimensional reconstruction, which collectively confirmed significant alterations in both developmental stages of the parasite.

**Figure 1:**
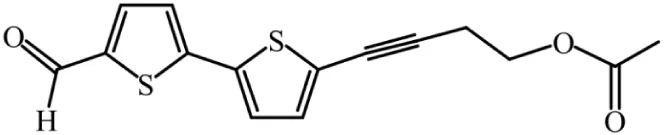
Chemical structure of 4-(5′-Formyl-[2,2′-bithiophen]-5-yl)­but-3-yn-1-yl acetate (**BT-Ac**).^22^

## 2 Methodology

### 2.1 Chemicals

Actinomycin D, carbonyl cyanide *m*-chlorophenylhydrazone (CCCP), digitonin, dimethyl sulfoxide (DMSO), folic acid, monodansylcadaverine (MDC), potassium cyanide (KCN), 2′,7′-dichlorofluorescin diacetate (H_2_DCFDA), rhodamine 123 (Rh123) and wortmannin were purchased from Sigma–Aldrich (St. Louis, MO, USA). Dulbecco’s modified Eagle’s medium (DMEM), fetal bovine serum (FBS), Annexin-V FITC, 3-(4,5-dimethylthiazol-2-yl)-2,5-diphenyltetrazolium bromide (MTT), propidium iodide (PI), 4-amino-5-methylamino-2″,7″-difluorofluorescein diacetate (DAFFM DA), 2,3-bis­(2-methoxy-4-nitro-5-sulfophenyl)-2*H*-tetrazolium-5-carboxanilide (XTT) and RNase A were obtained from Invitrogen (Grand Island, NY, USA). Roswell Park Memorial Institute medium (RPMI 1640) was obtained from Gibco (NY, USA). Uranyl acetate, sodium cacodylate, lead citrate, potassium ferrocyanide, glutaraldehyde, Polybed 81 resin and osmium tetroxide were bought from Electron Microscopy Sciences (EMS- Hatfield, USA). CellTiter-GLO kit viability assay was bought from Promega (Madison, Wisconsin, USA). All the reagents used were analytical grade. The defibrinated sheep blood was commercially obtained.

### 2.2 Compound

**BT-Ac** was synthesized as previously described by Machado et al, 2025.^22^ Stock solutions were aseptically prepared in DMSO and diluted in culture medium so that the DMSO concentration did not exceed 3% in the experiments.^24^

### 2.3 Cell Cultures

The experiments were performed with *L. amazonensis* (WHOM/BR/75/JOSEFA strain), isolated initially by C.A. Cuba–Cuba (Universidade de Brasília, Brazil) from a patient with diffuse cutaneous leishmaniasis. Promastigotes were cultured in Warren medium (brain heart infusion, hemin, and folic acid; pH 7.0) supplemented with 10% FBS and maintained at 25 °C. J774A.1 macrophages (sourced from Banco de Células do Rio de Janeiro, Rio de Janeiro, Brazil) were grown in RPMI-1640 medium (pH 7.2) supplemented with sodium bicarbonate, l-glutamine, and 10% FBS, incubated at 37 °C under a 5% CO_2_ atmosphere. Intracellular amastigotes of *L. amazonensis* were obtained by infecting macrophages with promastigotes and incubating them at 34 °C under a 5% CO_2_ atmosphere.

### 2.4 Antiproliferative Activity against *L. amazonensis*

i To determine the antiproliferative activity against the extracellular parasites, promastigotes in the logarithmic phase (1 × 10^6^ parasites/ml) were added in 96-well culture microplates at 25 °C in Warren’s medium supplemented with 10% FBS. Parasites were then incubated in the presence of different concentrations of **BT-Ac** (6.25–100 μM) and incubated for an additional 72 h. The activity was determined by the mitochondrial function, using the tetrazolium salt XTT at 0.5 mg/mL, as previously described.^25^ The absorbance was measured in a spectrophotometer (PowerWave XS , BIO-TEK, USA) at 450 nm. The inhibitory concentration for 50% of parasite growth (IC_50_) was calculated by nonlinear regression of data using GraphPad Prism 8 software.
ii To determine the antiproliferative activity against the intracellular parasites, J774A.1 macrophages at 5 × 10^5^ cells/mL and stationary promastigotes at 5 × 10^6^ cells/mL were seeded on sterile glass coverslips and incubated for 24 h at 34 °C under a 5% CO_2_ atmosphere. After 24 h of interaction, the wells were washed with RPMI 1640 medium to remove noninteriorized parasites. Afterward, the infected macrophages were treated with **BT-Ac** at different concentrations (6.25–200 μM) and incubated for 48 h. The percentage of infected macrophages was evaluated after Giemsa staining by microscopically counting the number of amastigotes per macrophage. The IC_50_ was calculated by nonlinear regression of data using GraphPad Prism 8 software.^26^

### 2.5 Cytotoxicity

To determine cytotoxicity in macrophage cells, J774A.1 macrophages at 5 × 10^5^ cells/mL were seeded in 96-well plates and incubated at 37 °C in a 5% CO_2_ air mixture for 24 h, for monolayer formation. After this, the macrophages were incubated for 48 h in the presence or absence of different concentrations of **BT-Ac** (50–1000 μM) and incubated for an additional 48 h at 37 °C in a 5% CO_2_ air mixture. The wells were then washed with PBS and the cell viability was measured by a colorimetric MTT assay as previously described.^27^ The absorbance was measured using a Bio-Tek PowerWave XS spectrophotometer at 570 nm, and the cytotoxic concentration for 50% of the cells (CC_50_) was determined by nonlinear regression of the data using GraphPad Prism 8 software.

### 2.6 Electron Microscopy

Ultrastructural and morphological alterations were assessed by transmission electron microscopy (TEM) and scanning electron microscopy (SEM), respectively. For this, promastigotes at a density of 1 × 10^6^ cells/mL were treated with 28.9 μM of **BT-Ac** and incubated for 72 h at 25 °C. For amastigote analysis, J774A.1 at 5 × 10^5^ cells/mL macrophages were previously infected with promastigotes at 5 × 10^6^ cells/mL as described above. The resulting macrophages infected with intracellular amastigotes were treated with **BT-Ac** at 50 μM and incubated for 48 h at 34 °C in a 5% CO_2_ atmosphere.

i For TEM the parasites were washed with PBS, fixed in 2.5% glutaraldehyde in 0.1 M sodium cacodylate buffer, and postfixed in a solution containing 1% osmium tetroxide, 0.8% potassium ferrocyanide, and 5 mM calcium chloride. The parasites were dehydrated in an acetone series and embedded in Polybed 81 resin for 72 h at 60 °C. Ultrathin sections were obtained in an ultramicrotome Leica EM UC7 and stained with 5% uranyl acetate and lead citrate. The images were taken in a JEOL JEM 1400 transmission electron microscope.^28^
ii For SEM, the parasites were washed with PBS, fixed in 2.5% glutaraldehyde in 0.1 M sodium cacodylate buffer, and placed on a specimen support with poly-*L*-lysine, dehydrated in a graded series of ethanol, critical-point dried in CO_2_, coated with gold, and observed in a dual-beam FEI-Scios scanning electron microscope. For amastigote analysis, mechanical macrophage membrane removal was performed using commercial tape strip.^28^

### 2.7 Mechanism of Action of **BT-Ac** on Parasites of *L. amazonensis*

For all subsequent mechanisms of action assays, parasites were exposed to their respective IC_50_ and 2 × IC_50_ concentrations. Promastigotes at a density of 1 × 10^6^ cells/mL were treated with concentrations of 28.9 μM and 57.8 μM of **BT-Ac** and incubated for 24 h at 25 °C.

For amastigote analysis, J774A.1 at 5 × 10^5^ cells/mL macrophages were previously infected with promastigotes at 5 × 10^6^ cells/mL as described in the Section 2.4. The resulting macrophages infected with intracellular amastigotes were treated with **BT-Ac** at 50 μM and 100 μM concentrations and incubated for 24 h at 34 °C in a 5% CO_2_ atmosphere. Cells were harvested using a cell scraper, the volume was aspirated and dispensed under pressure using a 30-gauge needle and a syringe to induce macrophage lysis. Amastigotes were subsequently separated by differential centrifugation at 1000 rpm for 5 min. The supernatant containing the amastigotes was carefully collected, counted in a Neubauer chamber, and 1 × 10^6^ cells/mL were used for further analysis.^26^

The effect of **BT-Ac** on parasites was measured in a flow cytometer or microplate reader. A FACSCalibur (Becton Dickinson, Rutherford, NJ, USA) flow cytometer equipped with CellQuest software (Joseph Trotter, Scripps Research Institute, La Jolla, CA, USA) was used and a total of 30,000 events were acquired in the region previously established as the one that corresponded to the parasites. The microplate reader used was Victor X3 (PerkinElmer, EUA).

After the incubation period, the parasites were washed with PBS and the following procedures were performed for each assay:

i Reactive oxygen species (ROS): the parasites were incubated with 10 μM of H_2_DCFDA in absence of light for 45 min.^29^ Fluorescence was detected in a flow cytometer. Hydrogen peroxide (H_2_O_2_) 50 μM treatment for 24 h was used as positive control.
ii Nitric oxide (NO): the parasites were incubated with DAF-FM DA 1 μM for 30 min at 37 °C.^30^ Then, the samples were rewashed, and incubated for an additional 15 min. Fluorescence was detected in a flow cytometer. H_2_O_2_ 50 μM treatment for 24 h was used as positive control.
iii Intracellular ATP: the Cell-title GLO assay kit was used to measure ATP production, following manufacturer’s instructions.^31^ Briefly, equal volumes were added in a 96 white well-plates and the luminescence was measured in a microplate reader. Potassium cyanide (KCN) 500 μM treatment for 5 min was used as a positive control.
iv. Mitochondrial membrane potential: the parasites were incubated with Rh123 for 15 min at 37 °C. Afterward, the parasites were rewashed and incubated for an additional 30 min at 37 °C.^32^ The fluorescence was detected in a flow cytometer and CCCP 100 μM treatment for 24 h was used as a positive control.
v. Lipid peroxidation: the parasites were incubated with 50 μM of DPPP for 15 min at room temperature in absence of light. The fluorescence of oxidized DPPP-O was determined in a microplate reader at λ_ex_ of 355 nm and λ_em_ of 460 nm.^33^ H_2_O_2_ 50 μM treatment for 24 h was used as positive control.
vi. Lipid bodies accumulation: the parasites were incubated with 10 mg/mL of Nile red for 30 min at room temperature in absence of light. The fluorescence was determined in a microplate reader at λ_ex_ of 485 nm and λ_em_ of 535 nm. H_2_O_2_ 50 μM treatment for 24 h was used as positive control.
vii. Membrane integrity: the parasites were incubated with 0.2 mg/mL of PI for 10 min at 25 °C.^34^ The fluorescence was detected in a flow cytometer, and digitonin 40 μM treatment for 5 min was used as a positive control.
viii. Cell size: no fluorescent probe was used. The cell size was assessed by a flow cytometer and actinomycin D 20 μM treatment for 24 h was used as a positive control.^35^
ix. Cell cycle: the parasites were fixed in iced methanol/PBS 70% for 24 h at 4 °C and under constant agitation. Then, the samples were rewashed with PBS and incubated with 20 μg/mL of PI and RNase for 45 min at 37 °C in the absence of light.^36^ The fluorescence was detected using a flow cytometer, and a 10 μM taxol treatment for 24 h was used as a positive control.
x. Autophagic vacuoles: the parasites were incubated with 0.05 mM of MDC for 1 h at 37 °C. Then, the samples were rewashed with PBS and the fluorescence was read in a microplate reader at λ_ex_ of 335 nm and λ_em_ of 518 nm.^37^ In parallel, the parasites were pretreated with wortmannin (0.5 μM) for 24 h, a potent inhibitor of PI3 kinase and consequently autophagic vacuole formation.^37^ PBS 25% was used as positive control.
xi. Acid vacuoles: the parasites were incubated with 10 mg/mL of acridin orange for 10 min at 25 °C. Then, the samples were rewashed with PBS and the fluorescence was detected in a flow cytometer. H_2_O_2_ 50 μM treatment for 24 h was used as positive control.^38^
xii. Phosphatidylserine exposure: the parasites were incubated with 5 μL of Annexin V-FITC in 100 μL of binding buffer (140 mM NaCl, 5 mM CaCl2, 10 mM HEPES-Na, pH 7.4) for 15 min at 25 °C.^39^ Then, 400 μL of binding buffer and PI (2 μg mL^–1^) were added. The fluorescence was detected in a flow cytometer, and miltefosine 40 μM treatment was used as a positive control.^40^

### 2.8 Nitric Oxide Assessment in J774A.1 Macrophages

J774A.1 macrophages at 5 × 10^5^ cells/mL, infected or not with amastigotes of *L. amazonensis* (at the same conditions as mentioned in Section 2.4), were treated with **BT-Ac** at 50 μM and 100 μM concentrations and incubated for 24 h at 34 or 37 °C in a 5% CO_2_ atmosphere. After the incubation period, cells were harvested using a cell scraper and stained with DAF-FM-DA at 1 μM and incubated for 30 min in the absence of light. Then, the cells were washed with PBS and the DAF-FM fluorescence was measured at λex of 495 nm and λem of 515 nm.^30^ H_2_O_2_ (50 μM) was used as positive control.

### 2.9 Time-Dependent Electron Microscopy

For time-dependent analysis, promastigotes were prepared for EM as previously described. Briefly, promastigotes at the density of 1 × 10^6^ cell/mL were treated with 28.9 μM of **BT-Ac** for 2, 4, 8, 12, 24, 36, 48, or 72 h and followed the steps described in 2.6.

### 2.10 Three-Dimensional Reconstruction

For FIB-SEM analysis, the samples were prepared following the same protocol used for TEM described in Section 2.6. After polymerization, aiming to create a pyramid on the top of the sample, the epoxy block was trimmed using a standard diamond knife. The block was mounted in a SEM stub with silver paint glue and coated with gold and the imaging was performed using a dual-beam FEI featuring a gallium ion source for focused-ion-beam milling. SEM imaging utilized a field emission gun and an in-lens secondary electron detector. Cross-sectional cutting was carried out with ion beam currents of 2.5 nA. Backscattered electron images were captured at an accelerating voltage of 2 kV and a beam current of 0.2 nA using the immersion lens mode with a CBS (Concentric BackScatter) detector. A series of images was collected in “slice-and-view” mode at 25,000× magnification, with a pixel size of 3072 and a milling step size of 5 nm. Following image acquisition, the contrast of the backscattered electron images was inverted to resemble the appearance of conventional TEM images. The alignment and rendering were performed using Amira software.

### 2.11 Statistical Analysis

The data are expressed as the means and standard deviations from at least three independent experiments. The data were analyzed using one- or two-way analysis of variance (ANOVA), with significant differences among means identified by Tukey or Dunnet tests. Values of *p* < 0.05 were considered statistically significant. The statistical analyses were performed using Prism 8 software (GraphPad, San Diego, CA, USA).

## 3 Results

### 3.1 Biological Activity

The antiproliferative activity assessed by the XTT assay demonstrated that **BT-Ac** can effectively inhibit the parasite growth with IC_50_ of 28.9 μM after 72 h of treatment against promastigotes, and IC_50_ of 50 μM against amastigotes. In contrast, cytotoxicity evaluated by the MTT assay showed a high CC_50_ value of 234.7 μM for macrophages. Accordingly, the IC_50_/CC_50_ ratio yielded a selectivity index value of 4.69 (Table 1), indicating **BT-Ac** is at least approximately 4.7 times more selective for parasites than host-cells.

**Table 1:** Antiproliferative Activity of **BT-Ac** on Forms of *Leishmania amazonensis* and Cytotoxicity on Macrophages J774A.1^a^

*Leishmania amazonensis*			
Promastigote IC_50_(μM)	Amastigote IC_50_ (μM)	Macrophage J774A.1 **CC**_**50**_ **(μM)**	SI* (amastigota)
28.9 ± 1.8	50.0 ± 0.66	234.7 ± 2.4	4.69

a Selectivity index: the ratio between CC_50_ and IC_50_. IC_50_: Inhibitory concentration of 50% of parasites; CC_50_: Cytotoxic concentration for 50% of host cells.

### 3.2 Oxidative Stress

Aiming to analyze the alterations caused by **BT-Ac** on parasites of *L. amazonensis*, the first variable evaluated was oxidative stress. A significant increase in total ROS in promastigotes after 24 h of treatment with **BT-Ac** was detected (Figure 2A1). In contrast, in amastigotes, the difference in total ROS was not significant (Figure 2A2) when compared to the control groups. This ROS increase in promastigotes was dose-dependent, showing 1.59- and 2.56-fold elevations following treatment with IC_50_ and 2 × IC_50_, respectively. Cells treated with H_2_O_2_ exhibited 3.76- and 2.92-fold increases in promastigotes and amastigotes, respectively (Figure 2A).

**Figure 2:**
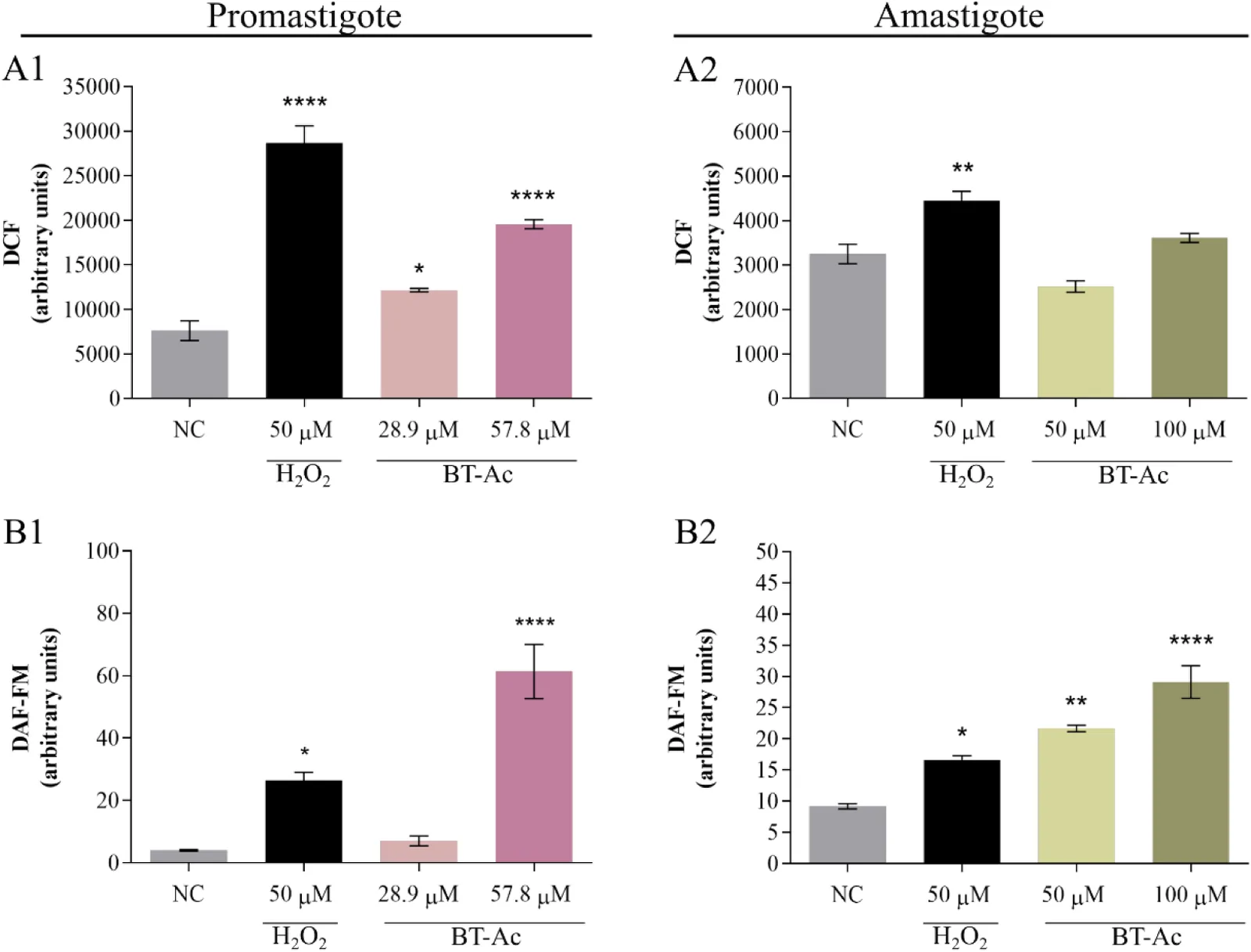
Free radical production in *Leishmania amazonensis* parasites treated with IC_50_ and 2 × IC_50_ for 24 h. (A1) Reactive oxygen species in promastigotes (pink) following treatment with 50 μM of H_2_O_2_, 28.9 μM and 57.8 μM of **BT-Ac**. (A2) Reactive oxygen species in amastigotes (green) following treatment with 50 μM of H_2_O_2_, 50 μM and 100 μM of **BT-Ac**. (B1) Reactive nitrogen species in promastigotes (pink) following treatment with 50 μM of H_2_O_2_, 28.9 μM, and 57.8 μM of **BT-Ac**. (B2) Reactive nitrogen species in amastigotes (green) following treatment with 50 μM of H_2_O_2_, 50 μM, and 100 μM of **BT-Ac**. The data are expressed as the mean fluorescence (in arbitrary units) ± SD of at least three independent experiments. *p < 0.05, **p < 0.01 ***p < 0.005, ****p ≤ 0.0001, a significant difference compared to the negative control (NC) group (i.e., untreated parasites). Statistical analysis was performed using one-way ANOVA and Tukey’s multiple comparisons tests (*n* = 3).

A significant 15.31-fold increase in intracellular nitric oxide production was observed in promastigotes treated with 2 × IC_50_ of **BT-Ac** after 24 h of treatment, compared to the control group (Figure 2B1). In amastigotes, a significant dose-dependent increase in nitric oxide production was observed, showing 2.36- and 3.17-fold elevations compared to the control group (Figure 2B2). Cells treated with H_2_O_2_ exhibited a significant increase in nitric oxide production of 6.63- and 1.82-fold in promastigotes and amastigotes, respectively (Figure 2B).

### 3.3 Mitochondrial Dysfunction and Structural Changes

Along with structural changes, mitochondrion showed dysregulation. The micrographs revealed an intense membrane disorganization, with an unprecedented arrangement between the subpellicular microtubules and the membrane interface, as well as lipid droplets accumulation (Figure 3A2–A4). Besides, nuclear disorganization, intense exocytic activity and mitochondrial swelling were observed in both evolutionary forms, treated with **BT-Ac** (Figure 3A2–A4 for promastigotes and Figure 3D2–D4 for amastigotes) if compared to the untreated group (Figure 3A1 and 3E1). Aligned with mitochondria swelling, a significant increase in intracellular ATP in promastigotes treated with **BT-Ac** after 24 h of treatment was observed compared to the control group. This increase was approximately 2.36- and 2.25-fold following treatment with IC_50_ and 2 × IC_50_, respectively (Figure 3B). In amastigotes, a 1.16-fold decrease in intracellular ATP levels was observed when treated with the IC_50_ of **BT-Ac**, whereas a 1.10-fold increase was detected with 2 × IC_50_ treatment (Figure 3E). The cytochrome oxidase complex inhibitor KCN was used as a positive control and induced a significant reduction in intracellular ATP levels, with decreases of 2.11- and 1.37-fold in promastigotes and amastigotes, respectively (Figure 3B and 3E).

**Figure 3:**
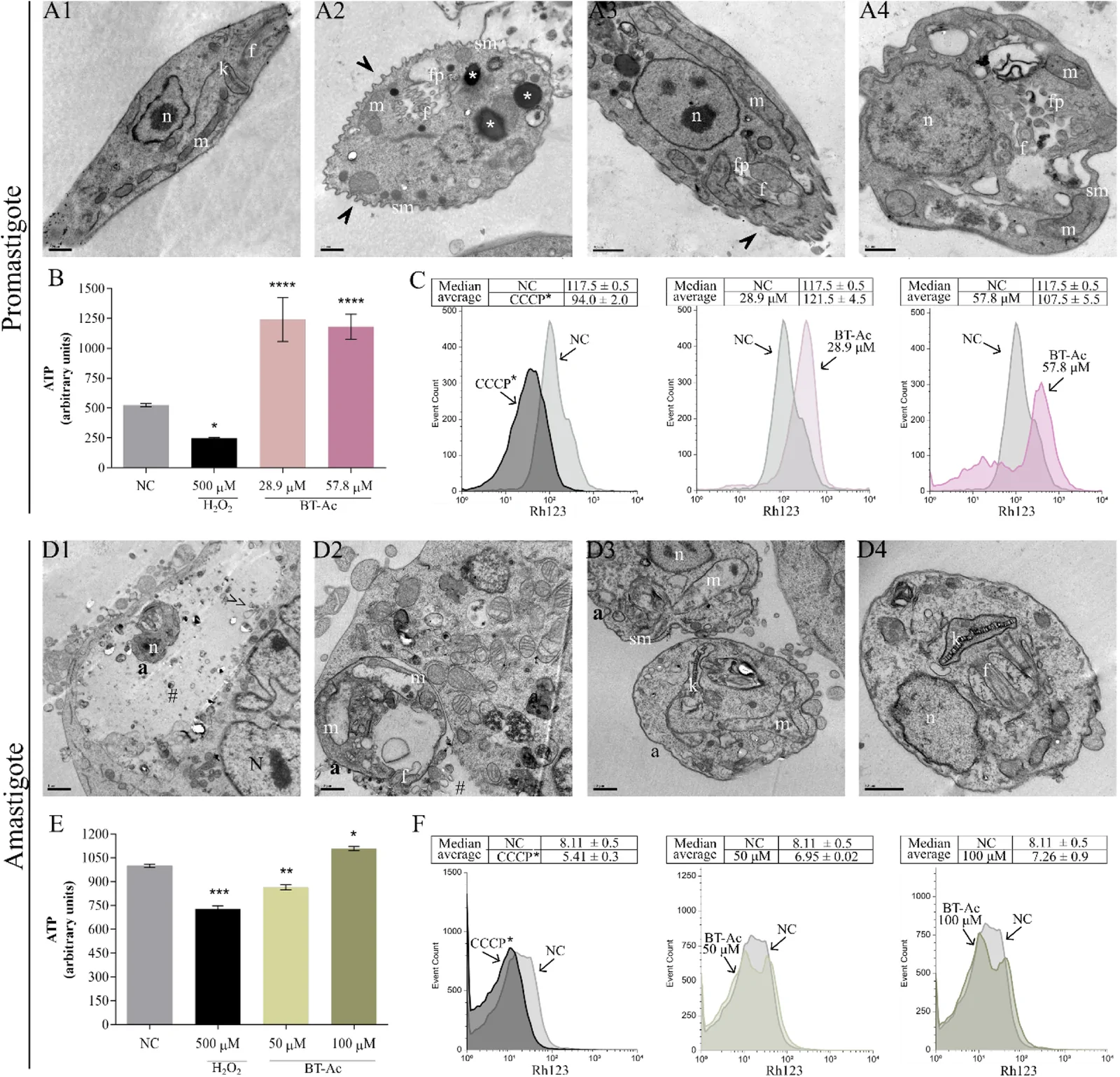
Ultrastructural characteristics and mitochondrion function in *Leishmania amazonensis* parasites treated with IC_50_ and 2 × IC_50_ for 24 h. (A) Transmission electron microscopy of (A1) untreated promastigotes; (A2–A4) promastigotes treated with 28.9 μM of **BT-Ac**. (B) Total intracellular ATP of promastigotes following treatment with 500 μM of KCN, 28.9 μM, and 57.8 μM of **BT-Ac**. (C) Mitochondrial membrane potential of promastigotes following treatment with 100 μM of CCCP, 28.9 μM, and 57.8 μM of **BT-Ac**. (D) Transmission electron microscopy of (D1) untreated amastigotes; (D2–D4) amastigotes treated with 50 μM of **BT-Ac**. (E) Total intracellular ATP of amastigotes following treatment with 500 μM of KCN, 50 μM, and 100 μM of **BT-Ac**. (F) Mitochondrial membrane potential of amastigotes following treatment with 100 μM of CCCP, 50 μM, and 100 μM of **BT-Ac**. The data are expressed as the mean fluorescence or luminescence (in arbitrary units) ± SD of at least three independent experiments. *p < 0.05, **p < 0.01 ***p < 0.005, ****p ≤ 0.0001, a significant difference compared to the negative control (NC) group (i.e., untreated parasites). Statistical analysis was performed using one-way ANOVA and Tukey’s multiple comparisons tests (*n* = 3). (a) Amastigote; (m) mitochondria; (n) nucleus; (k) kinetoplast; (f) flagellum; (fp) flagellar pocket; (sm) subpellicular microtubules; (N) macrophage nucleus; (*) lipid bodies; (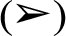) disordered microtubules. Scale bar: 1 μm = D1. 0.5 μm = A1–A4 and D2–D4.

Despite the alterations mentioned before, histograms showed no significant alterations at the mitochondrial membrane potential. The mitochondrial uncoupler CCCP was used as positive control and resulted in a reduction of Rh123 fluorescence by 1.25-fold in promastigotes and 1.50-fold in amastigotes (Figure 3C and 3F). Both decreases in ΔΨm indicate mitochondrial depolarization.

### 3.4 Lipid Content Alterations

As pointed out before, the accumulation of lipid vacuoles in promastigotes (Figure 4A2–A4) is more evident in these micrographs compared to the untreated control group (Figure 4A1). Lipid peroxidation was assessed by spectrofluorometry using DPPP. Promastigotes treated with IC_50_ and 2 × IC_50_ of **BT-Ac** for 24 h exhibited a significant increase in lipid peroxidation of 3.38- and 3.29-fold, respectively, compared to the control group (Figure 4B). In contrast, in amastigotes, a significant increase of 1.33-fold was observed only after treatment with 2 × IC_50_ of **BT-Ac** (Figure 4F). In promastigotes, treatment with H_2_O_2_ induced a 3.52-fold increase in lipid peroxidation and in amastigotes the increase was 1.24-fold (Figure 4B and 4F).

**Figure 4:**
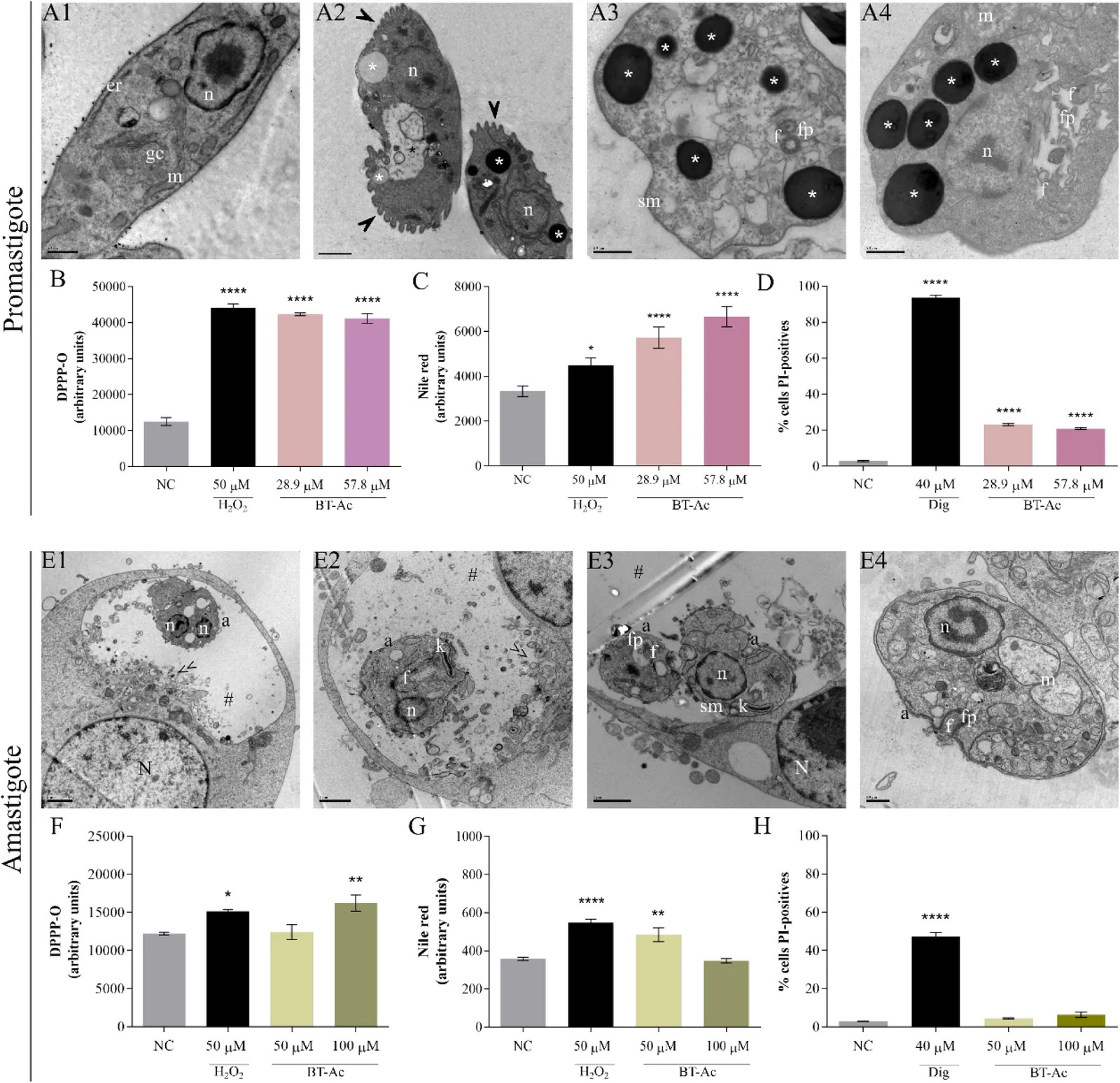
Lipid content in *Leishmania amazonensis* parasites treated with IC_50_ and 2 × IC_50_ for 24 h. (A) Transmission electron microscopy of (A1) untreated promastigotes; (A2–A4) promastigotes treated with 28.9 μM of **BT-Ac**. (B) Lipoperoxidation in promastigotes following treatment with 50 μM of H_2_O_2_, 28.9 μM, and 57.8 μM of **BT-Ac**. (C) Lipid inclusions in promastigotes following treatment with 50 μM of H_2_O_2_, 28.9 μM, and 57.8 μM of **BT-Ac**. (D) Membrane integrity of promastigotes following treatment with 40 μM of digitonin, 28.9 μM, and 57.8 μM of **BT-Ac**. (E) Transmission electron microscopy of (E1) untreated amastigotes; (E2–E4) amastigotes treated with 50 μM of **BT-Ac**. (F) Lipoperoxidation in amastigotes following treatment with 50 μM of H_2_O_2_, 50 μM, and 100 μM of **BT-Ac**. (G) Lipid inclusions in amastigotes following treatment with 50 μM of H_2_O_2_, 50 μM, and 100 μM of **BT-Ac**. (H) Membrane integrity of amastigotes following treatment with 40 μM of digitonin, 50 μM, and 100 μM of **BT-Ac**. The data are expressed as the mean fluorescence (in arbitrary units) ± SD of at least three independent experiments. *p < 0.05, **p < 0.01, ***p < 0.005, ****p ≤ 0.0001, a significant difference compared to the negative control (NC) group (i.e., untreated parasites). Statistical analysis was performed using one-way ANOVA and Tukey’s multiple comparisons tests (*n* = 3). (a) Amastigote; (m) mitochondria; (n) nucleus; (k) kinetoplast; (f) flagellum; (fp) flagellar pocket; (sm) subpellicular microtubules; (N) macrophage nucleus; (*) lipid bodies; (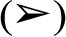) disordered microtubules. Scale bar: 1 μm = E1–E2; 0.5 μm = A1–A4 and E3–E4.

To quantify lipid bodies in the parasites, Nile red fluorescent dye was used due to its affinity for neutral lipids. The increase in the number of lipid vacuoles in promastigotes treated with **BT-Ac** was significantly higher compared to untreated parasites (Figure 4C), showing 1.71- and 1.99-fold increases after treatment with IC_50_ and 2 × IC_50_, respectively, whereas amastigotes only showed a significant increase in lipid vacuole number after IC_50_ **BT-Ac** treatment with an increase of 1.35-fold (Figure 4G). Parasites treated with H_2_O_2_ exhibited a significant increase in the presence of lipid bodies, with 1.35-fold in promastigotes and 1.62-fold in amastigotes compared to the control (Figure 4C and 4G). These data corroborate the results obtained from the time-dependent analysis on promastigotes performed by electron and fluorescence microscopy.

As lipid content was found altered, PI was used to assess the integrity of parasite cell membranes. The data show an increase in PI fluorescence intensity in promastigotes treated with IC_50_ and 2 × IC_50_ of **BT-Ac** for 24 h with a significant increase of 8.15- and 7.35-fold after IC_50_ and 2 × IC_50_ respectively (Figure 4D), indicating plasma membrane disruption, whereas in amastigotes, no significant increase in PI fluorescence was observed (Figure 4H), indicating that amastigote membrane integrity was not affected by **BT-Ac**. Cells treated with digitonin had their membrane integrity compromised, showing increases of 32.96- and 15.80-fold in promastigotes and amastigotes, respectively (Figure 4D and 4H).

### 3.5 Cell Cycle and Morphology Changes

SEM revealed morphological alterations in **BT-Ac**–treated promastigotes, including cell body rounding, leakage of cytoplasmic contents, and reduction in flagellum length (Figure 5A3–A4). Untreated parasites, on the other hand, displayed an elongated body with a flagellum proportional to the cell size and a smooth, continuous surface (Figure 5A1–A2). In amastigotes, we only observed the reduction of number of parasites per macrophages, as no morphological alteration was observed (Figure 5D1–D4). Cell size was evaluated by flow cytometry, and the bar graphs show a slight but significant reduction in the size of promastigotes, approximately 1.10-fold, after 24 h of treatment with IC_50_ and 2 × IC_50_ of **BT-Ac** compared to the control group (Figure 5B). In amastigotes, a 1.78-fold increase in cell volume was observed after treatment with IC_50_ of **BT-Ac**, followed by a decrease in total cell volume after treatment with 2 × IC_50_ of **BT-Ac**; however, this reduction was not significant compared to the control (Figure 5E). Cells treated with actinomycin D showed a significant reduction of 2.54- and 1.62-fold in promastigotes and amastigotes, respectively (Figure 5B and 5E).

**Figure 5:**
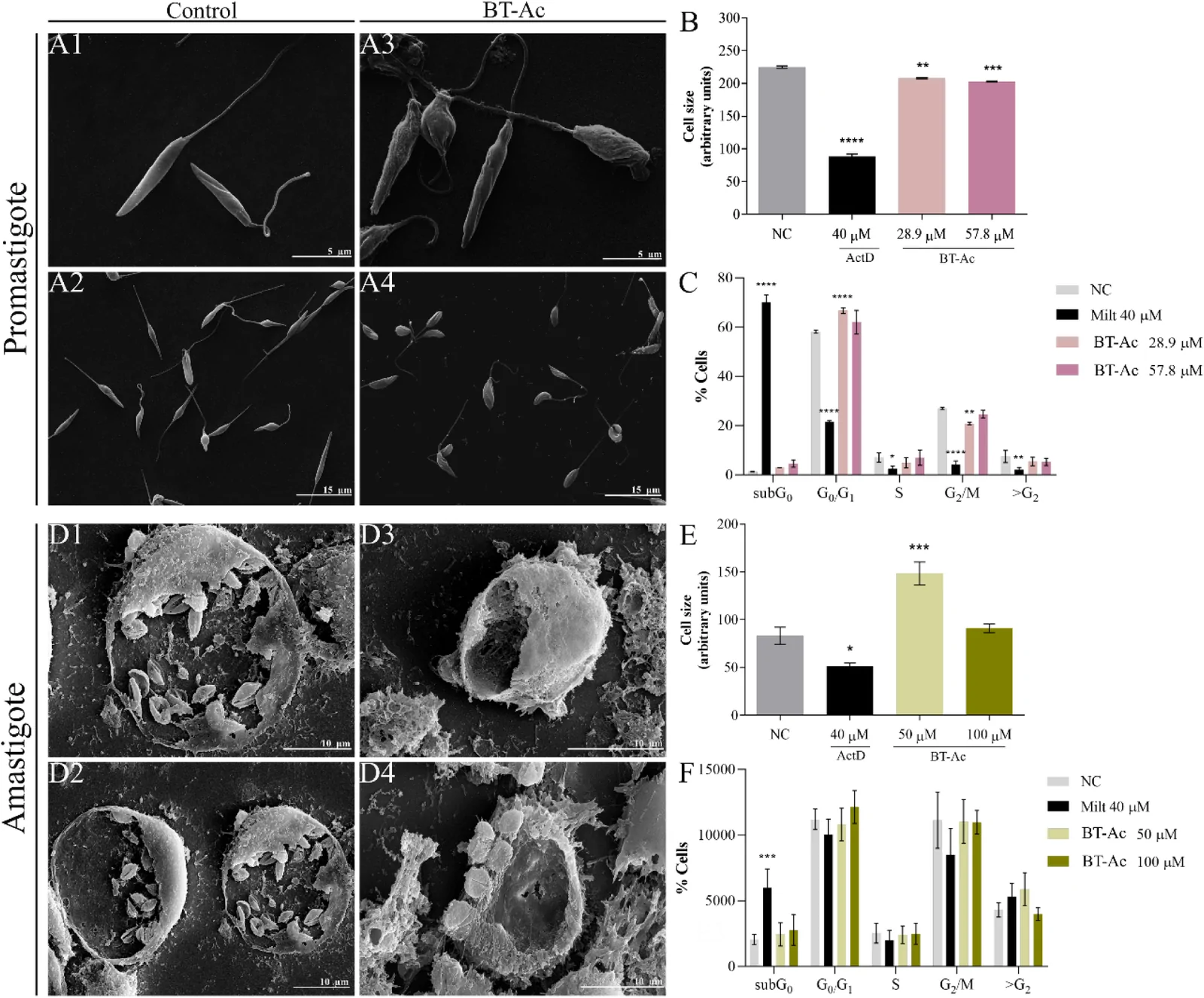
Morphology and cell cycle of *Leishmania amazonensis* parasites treated with IC_50_ and 2 × IC_50_ for 24 h. (A) Scanning electron microscopy of (A1–A2) untreated promastigotes; (A3–A4) promastigotes treated with 28.9 μM of **BT-Ac**. (B) Cell size of promastigotes after treatment with 40 μM of actinomycin D, 28.9 μM, and 57.8 μM of **BT-Ac**. (C) Cell cycle of promastigotes after treatment with 40 μM of miltefosine, 28.9 μM, and 57.8 μM of **BT-Ac**. (D) Scanning electron microscopy of (D1–D2) untreated amastigotes; (D3–D4) amastigotes treated with 50 μM of **BT-Ac**. (E) Cell size of amastigotes after treatment with 40 μM of actinomycin D, 50 μM, and 100 μM of **BT-Ac**. (F) Cell cycle of amastigotes after treatment with 40 μM of miltefosine, 50 μM, and 100 μM of **BT-Ac**. The data are expressed as the mean fluorescence (in arbitrary units) ± SD of at least three independent experiments. *p < 0.05, **p < 0.01 ***p < 0.005, ***p ≤ 0.0001, a significant difference compared to the negative control (NC) group (i.e., untreated parasites). Statistical analysis was performed using one-way or two-way ANOVA and Tukey’s multiple comparisons tests (*n* = 3). Scale bar: 5 μm = A1 and A3; 10 μm = D1–D4; 15 μm = A2 and A4.

The cell cycle was evaluated by flow cytometry to assess changes in the DNA content of the parasites through PI fluorescence intensity. Miltefosine, used as a positive control, notably induced cell cycle arrest in the sub-G0 phase in both promastigotes and amastigotes, as evidenced by a significant increase in the number of <2n cells, indicating DNA fragmentation. Treatment with **BT-Ac** resulted in a slight accumulation of cells in the G0/G1 phase (2n) in promastigotes (Figure 5C) with a significant increase of 1.15-fold in cells at this phase, and a decrease in the number of cells in the S and G2/M phases (2n–4n and 4n) with a significant reduction of 1.30-fold of cells. In amastigotes, no differences in cell cycle phases were observed, indicating that the tested compound does not alter the cell cycle of *L. amazonensis* amastigotes (Figure 5F).

### 3.6 Cell Death Pathway Evaluation

The fluorescent probe MDC, which accumulates in acidic vacuoles, was used to evaluate autophagic vacuoles, while wortmannin was used to prevent autophagy. Data showed that after 24 h of treatment with IC_50_ and 2 × IC_50_ of **BT-Ac** without wortmannin, there was a significant increase in acid vacuole formation in promastigotes of 2.86- and 20.35-fold (Figure 6A1) and in amastigotes of 1.38- and 1.58-fold, respectively (Figure 6A2), represented by increased MDC fluorescence compared to control groups. Positive controls showed increases of 2.06- and 1.22-fold in promastigotes and amastigotes, respectively (Figure 6A1 and A2) in the group without wortmannin. However, only in promastigotes was the autophagy inhibitor—wortmannin able to prevent autophagic vacuole formation, resulting in decreases of 2.57-, 5.90-, and 1.94-fold after treatment with positive control, IC_50_, and 2 × IC_50_, respectively (Figure 6A1), indicating that some of those acid vacuoles were in fact autophagic.

**Figure 6:**
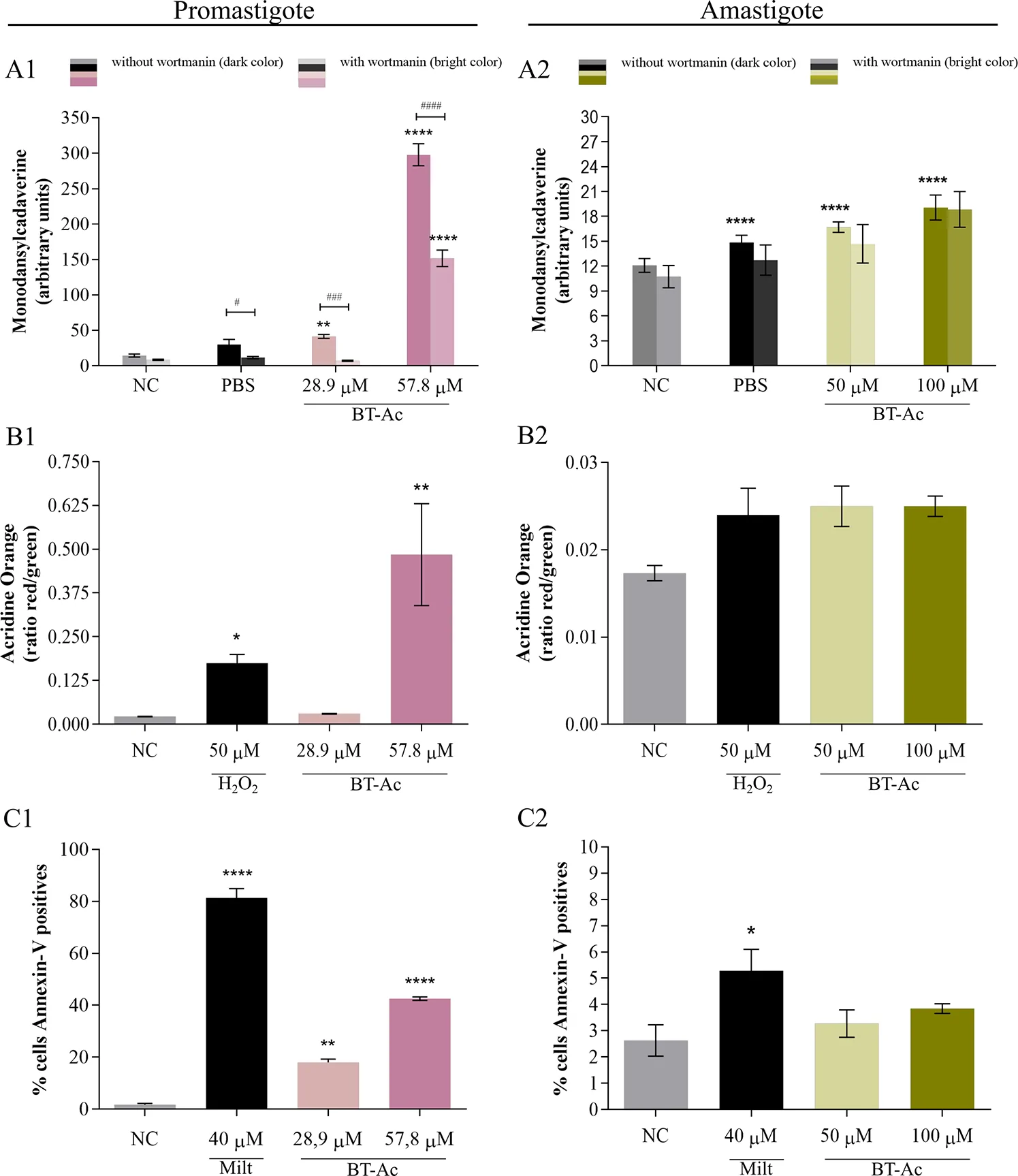
Cell death of *Leishmania amazonensis* parasites treated with IC_50_ and 2 × IC_50_ for 24 h. (A) Autophagic vacuoles (A1) in promastigotes (pink) after treatment with PBS 25%, 28.9 μM and 57.8 μM of **BT-Ac** and (A2) in amastigotes (green) after treatment with PBS 25%, 50 μM and 100 μM of **BT-Ac**. (B) Acid vacuoles (B1) in promastigotes after treatment with 50 μM of H_2_O_2_, 28.9 μM, and 57.8 μM of **BT-Ac** and (B2) in amastigotes after treatment with 50 μM of H_2_O_2_, 50 μM, and 100 μM of **BT-Ac**. (C) Phosphatidylserine exposure (C1) in promastigotes after treatment with 40 μM of miltefosine, 28.9 μM, and 57.8 μM of **BT-Ac** and (C2) in amastigotes after treatment with 40 μM of miltefosine, 50 μM, and 100 μM of **BT-Ac**. Statistical analysis was performed using one-way or two-way ANOVA and Tukey’s multiple comparisons tests (*n* = 3). *p < 0.05, **p < 0.01 ***p < 0.005, ****p ≤ 0.0001, a significant difference compared to the negative control (NC) and without wortmannin; ^#^ *p* < 0.05, ^##^ *p* < 0.01 ^###^ *p* < 0.005, ^####^ *p* ≤ 0.0001, a significant difference compared to the group without wortmannin.

On the other hand, the presence of general acidic vacuoles was evaluated by flow cytometry through the fluorescence of the acridine orange dye. The bar graph shows that only in promastigotes was there an increase in the red/green fluorescence ratio, representing a significant increase in acidic vacuoles of 7.95- and 22.03-fold after treatment with H_2_O_2_ and IC_50_, respectively (Figure 6B1). In amastigotes, no significant acid vacuoles were detected by acridine orange staining (Figure 6B2).

To analyze apoptosis-like cell death, double staining with PI and Annexin-V were used. A significant increase of 10.76- and 25.37-fold in the percentage of Annexin-V positive promastigote cells was observed compared to the control, indicating that treatment with IC_50_ and 2 × IC_50_ of **BT-Ac** for 24 h induced phosphatidylserine exposure on the parasite membrane (Figure 6C1). In amastigotes, the increase in the percentage of Annexin-V positive cells was not significant (Figure 6C2). Parasites treated with miltefosine (Figure 6C1 and C2) showed a significant increase of 48.54- and 2.0-fold in Annexin-V positive cells in promastigotes and amastigotes, respectively. Phosphatidylserine is a phospholipid normally located on the inner leaflet of the plasma membrane under healthy conditions but becomes exposed on the surface of cells undergoing apoptosis, indicating that **BT-Ac** may have induced an apoptosis-like process in promastigote forms of *L. amazonensis*.

Nitric oxide production was also evaluated by flow cytometry using the DAF-FM DA probe in macrophages infected or uninfected with *L. amazonensis*. It was observed that nitric oxide production was increased in infected macrophages compared to uninfected ones, with this increase being even more pronounced after treatment with IC_50_ and 2 × IC_50_ of **BT-Ac**. Compared to the uninfected control, uninfected macrophages treated with H_2_O_2_ showed a significant 1.59-fold decrease in nitric oxide production, whereas infected macrophages treated with IC_50_ and 2 × IC_50_ of **BT-Ac** exhibited significant increases of 2.08- and 1.25-fold, respectively. H_2_O_2_ and IC_50_ treated infected macrophages, when compared to treated uninfected cells, showed significant increases in nitric oxide levels of 1.99- and 1.93-fold respectively (Figure 7).

**Figure 7:**
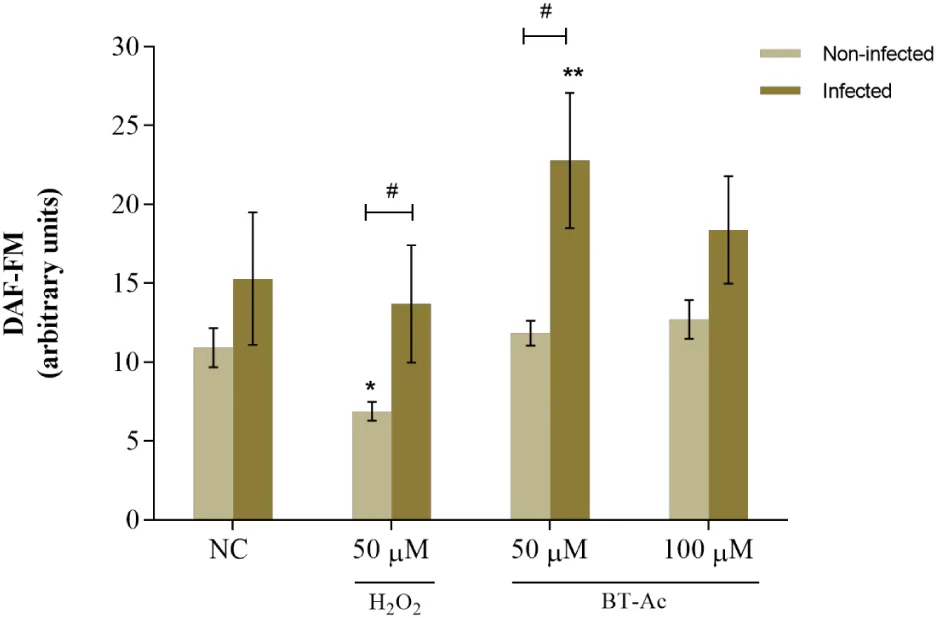
Nitric oxide in infected or noninfected J774A.1 macrophages after treatment with 50 μM of H_2_O_2_, 50 μM, and 100 μM of **BT-Ac**. The data are expressed as the mean fluorescence (in arbitrary units) ± SD of at least three independent experiments. *p < 0.05, **p < 0.01 ***p < 0.005, ****p ≤ 0.0001, a significant difference compared to the negative control (NC) and uninfected group (i.e., untreated parasites). ^#^ *p* < 0.05 a significant difference compared to the negative control (NC) and infected group. Statistical analysis was performed using one-way or two-way ANOVA and Tukey’s multiple comparisons tests (*n* = 3).

### 3.7 Further Ultrastructural Investigations

As mentioned before, unprecedented alterations in the membrane and subpellicular microtubules were detected in promastigotes after 72 h of treatment. To further investigate the onset of this structural disorganization, time-dependent imaging analyses were performed. Both SEM and TEM were employed to assess morphological alterations, while fluorescence microscopy was used to monitor the accumulation of lipid bodies. From 0 to 72 h, the alterations in the parasite membrane appeared to be time dependent; the longer the parasites were exposed to **BT-Ac**, the more pronounced the membrane shedding became (Figure 8). After 8 h of incubation, SEM and TEM revealed the initial signs of membrane wrinkling, which progressively became more evident (Figure 8A1–D9). The accumulation of lipid droplets also followed a time-dependent pattern; however, the presence of vacuoles was already observed as early as 4 h of incubation (Figure 8E1–F9).

**Figure 8:**
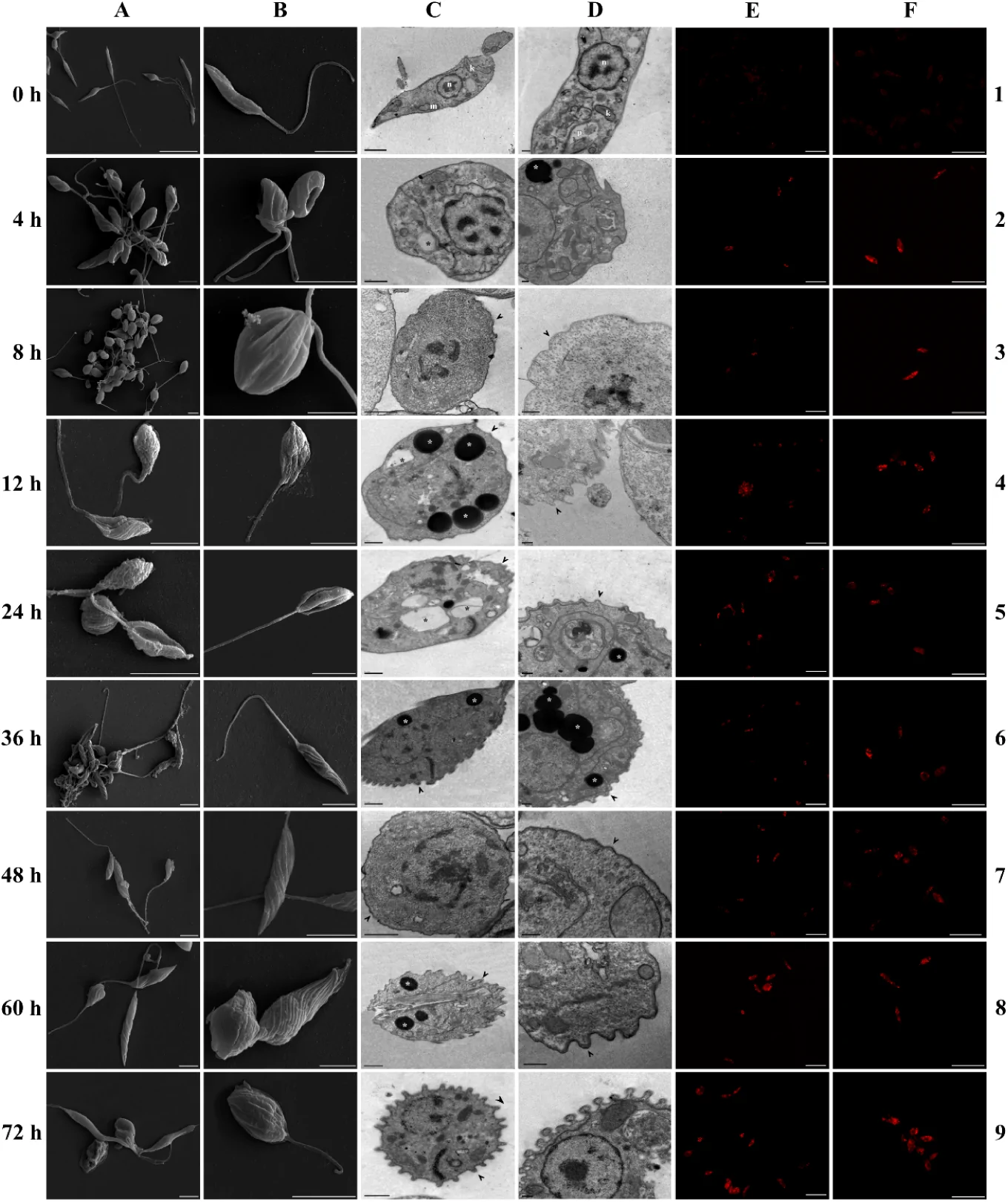
Ultrastructural and morphological alterations time-dependent in promastigote of *Leishmania amazonensis* treated with 28.9 μM of **BT-Ac** for 0 to 72 h. (A–B) Scanning electron microscopy; (C–D) transmission electron microscopy; (E–F) fluorescence microscopy of lipid bodies stained with Nile red. (n) nucleus; (m) mitochondrion; (k) kinetoplast; (p) flagellar pocket; (*) white: lipid body; (*) black: autophagic vacuole; (>) alterations in cell membrane. Bars = 5 μm: A1–A9, B1–B2, B4–B7; 2 μm: B3, B8 0.5 μm: C1–C9; 0.2 μm: D1–D9; 20 μm: E1–E9, F1–F9.

To rule out technical artifacts and confirm these alterations by an independent approach, three-dimensional reconstruction was performed on promastigotes treated with IC_50_ concentrations for 72 h. The virtual 3D model highlighted the alterations occurring at the subpellicular–membrane interface, as protrusions observed in treated samples (Figure 10) were absent in control cells (Figure 9). Another important observation is that the microtubule array closely followed the membrane curves, indicating the absence of vesicle or bleb formation, as clearly shown in Figure 10H. Moreover, SEM images revealed no evidence of exocytosis through the cell body or membrane detachment. Additionally, autophagic vacuoles and lipid droplets were also detected by serial sectioning (Figure 10G).

**Figure 9:**
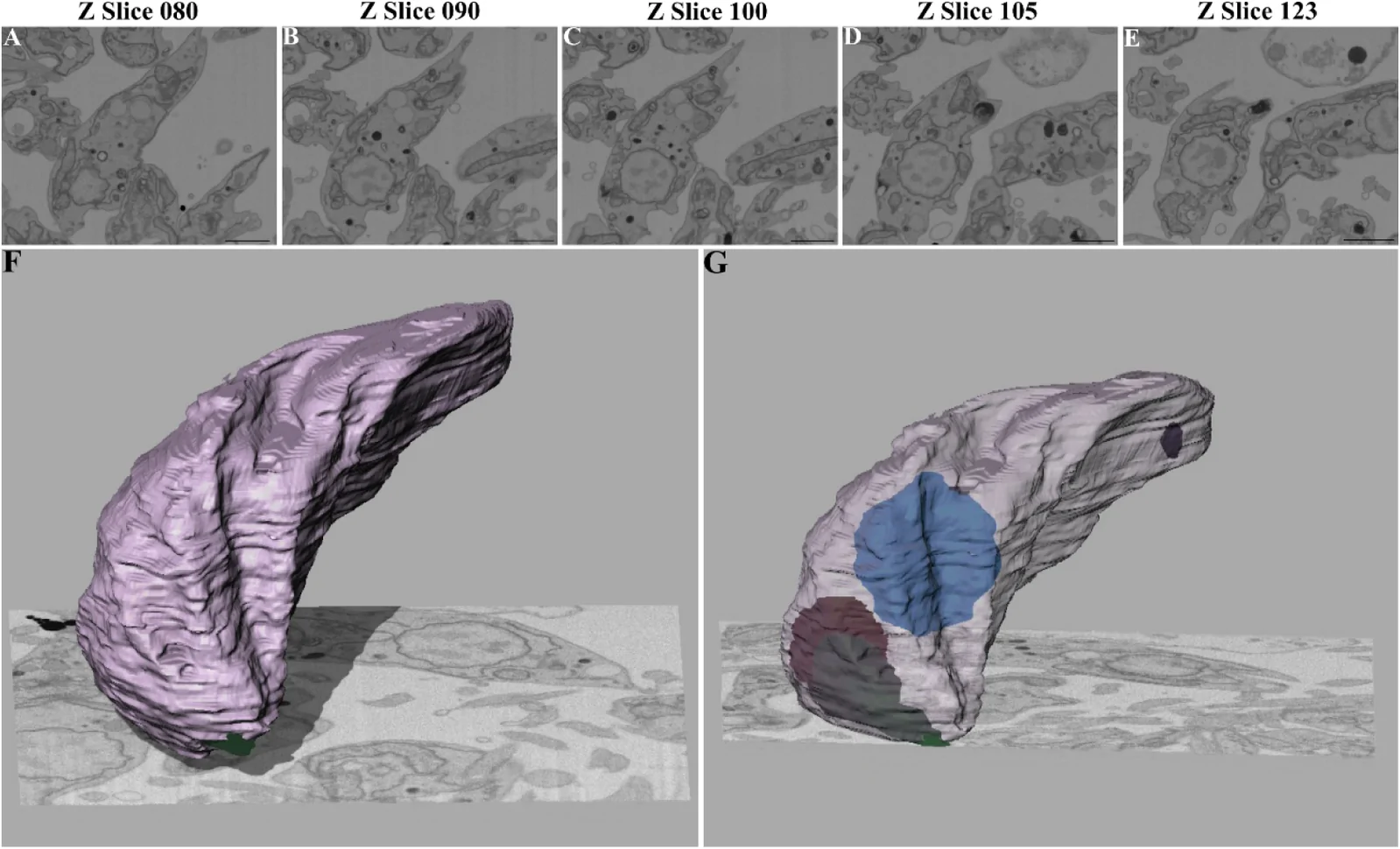
Three-dimensional reconstruction of promastigotes of *Leishmania amazonensis* untreated. Serial sections were obtained by dual-beam FIB-SEM (A–E) Representative sections of the image pack. (F–G) 3D virtual modeling by Amira software. Pink: membrane; blue: nucleus; purple: lipid bodies; green: flagellar pocket; red: kinetoplast. Scale bar: 3 μm = A–E.

**Figure 10:**
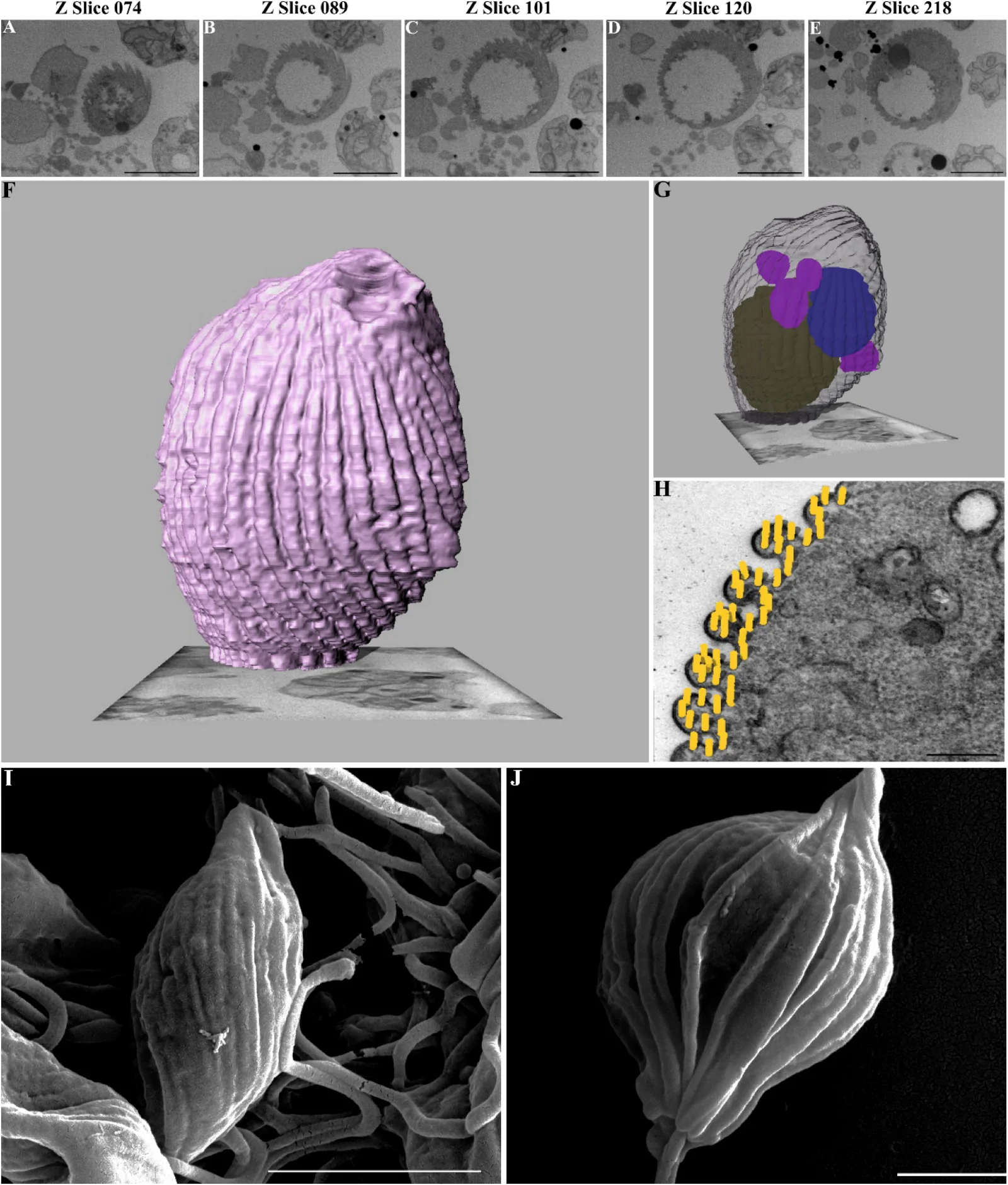
Three-dimensional reconstruction of promastigotes of *Leishmania amazonensis* treated with IC_50_ (28.9 μM) of **BT-Ac** for 72 h. Serial sections were obtained by dual-beam FIB-SEM (A–E) Representative sections of the image pack. (F–H) 3D virtual modeling by Amira software. (I–J) Scanning electron micrographs. Pink: membrane; blue: nucleus; purple: lipid bodies; green: autophagic vacuoles; yellow: microtubules. Scale bar: 4 μm = A–E; 3 μm = I; 2 μm = J; 200 nm H.

## 4 Discussion

It is noteworthy that some NTDs are more overlooked than others. Leishmaniasis fits this context, as its treatment relies almost exclusively on drugs that have existed for over 80 years and that exhibit high toxicity, parenteral administration, and potentially fatal adverse effects, while not always achieving clinical efficacy and parasitological cure.^20,41–43^ Despite recent advances, the treatment of leishmaniasis remains unsatisfactory. Considering that pharmaceutical industries show little interest in NTDs from a commercial standpoint, academic research remains the only alternative for the development of new methods that could be applied to the treatment of leishmaniasis.^41,44^

In literature, several studies have demonstrated the efficacy of thiophene derivatives against *Leishmania amazonensis*,^45^ *L. donovani*,^46^ and *L. infantum*.^47,48^ In addition to thiophenes, a scaffold variation, bithiophenes, has also shown promising activity against *L. amazonensis*.^21^ **BT-Ac**, a bithiophene derivative, was previously reported to display significant antitrypanosomal activity against *Trypanosoma cruzi*,^22^ triggering multiple cell death pathways in diverse life cycle stages of the parasite and presenting anti-inflammatory properties and good drugability.^23^ This finding motivated us to further investigate its potential against *L. amazonensis*. As expected, **BT-Ac** exhibited potent antiproliferative activity against *L. amazonensis* at nontoxic concentrations for macrophages, yielding a selectivity index of 4.69 against amastigotes. These results corroborate previous reports confirming the leishmanicidal activity of thiophenes.^49^

To elucidate the cell death pathways triggered by **BT-Ac** in *L. amazonensis* and to determine whether it shares a conserved mode of action among trypanosomatid parasites, we conducted electron microscopy (EM) and biochemical assays.

Treatment of *L. amazonensis* with **BT-Ac** triggered structural and biochemical events consistent with the induction of cell death. The first evidence of damage was revealed by electron microscopy (EM), a key approach for detecting cell death and evaluating parasite morphology, particularly in trypanosomatids, which possess distinctive ultrastructural features.^50–52^ SEM analyses showed early morphological alterations in promastigotes, including cell body rounding, cytoplasmic leakage, flagellum shortening, and irregularities on the plasma membrane—hallmarks often associated with oxidative stress. TEM analysis provided deeper insight, revealing nuclear and membrane disruption, lipid body accumulation, mitochondrial swelling, high exocytic activity, and autophagic vacuoles in both promastigotes and amastigotes. Similar patterns have been described in trypanosomatids under oxidative stress,^24,26,53^ reinforcing the link between **BT-Ac** exposure and the activation of oxidative damage pathways.

A likely initiating event is the overproduction of ROS and RNS, which we detected in both parasite forms after **BT-Ac** treatment. ROS are chemically reactive molecules formed by incomplete oxygen reduction, while RNS are nitric oxide–derived compounds; although both participate in normal cell regulation, excessive levels result in oxidative and nitrosative stress.^54–57^ These radicals readily react with proteins, lipids, and DNA, compromising macromolecular integrity and driving the accumulation of lipid bodies.^58^ The time-dependent analyses revealed that lipid bodies appear within hours of treatment, well before major membrane alterations become visible, suggesting that oxidative damage to lipids may be among the earliest consequences of **BT-Ac** action.

Lipid peroxidation, a direct outcome of ROS/RNS attack on membrane lipids, can impair membrane function and stability, leading to rupture^59^ and further contributing to lipid body accumulation.^58^ Consistent with this, our assays showed that membrane alterations began around 8 h post-treatment and intensified thereafter. This structural destabilization was accompanied by energy metabolism changes: ATP levels rose sharply in promastigotes and fluctuated biphasically in amastigotes (initial decrease followed by increase), a response that may represent a final cellular attempt to maintain homeostasis before mitochondrial depolarization.^60^

Lipid bodies are dynamic intracellular organelles composed of a core of neutral lipids surrounded by a phospholipid monolayer and associated proteins, which interact with other cellular components. The formation of these structures is a common cellular process, and their role is to function as an energy reservoir, but their accumulation has been associated with indicative of pathological degeneration caused, for example, by inhibition of the lipid synthesis pathway or by oxidative damage to lipids due to the effect of ROS.^60–62^ Other studies have also reported an increase in lipid body formation in trypanosomatids under stress conditions.^63,64^

The culmination of these events is the activation of multiple cell death pathways. Apoptotic-like features such as loss of plasma membrane integrity, cell shrinkage (pyknosis), and phosphatidylserine exposure were observed exclusively in **BT-Ac** treated promastigotes. Under physiological conditions, phosphatidylserine is confined to the inner leaflet of the membrane, but its externalization during apoptosis marks cells for phagocytosis.^65–67^ In parallel, autophagic vacuoles were present in both parasite forms, indicating activation of autophagy, a process that can be initially protective by degrading damaged components,^68,69^ but that in kinetoplastids can lead to cell death depending on the stress context.^70^ Inhibition experiments with wortmannin, a PI3K blocker essential for autophagosome maturation, reduced autophagic vacuoles only in promastigotes, suggesting that autophagy plays a more prominent role in promastigote death.^71,72^

In addition to autophagic vacuoles, other acidic cellular compartments such as acidocalcisomes were detected at increased levels in *L. amazonensis* promastigotes after **BT-Ac** treatment. Acidocalcisomes are spherical structures (0.2–0.6 μm) with an electron-lucent matrix, typically more abundant in amastigote forms, and are involved in ion storage (Ca^2+^, Mg^2+^, Na^+^, K^+^, Zn^2+^, Fe^2+^, phosphates), pH regulation, and osmoregulation via the contractile vacuole.^73^ Their increase may reflect a compensatory attempt to restore homeostasis in response to osmotic and energetic imbalance caused by **BT-Ac**.^74^

Cell cycle analysis revealed that **BT-Ac** did not alter the progression of *L. amazonensis* in either morphological form. However, macrophage activation was evident through increased nitric oxide production in infected cells relative to uninfected ones. Upon recognition of *Leishmania* spp., macrophages become “effector cells” capable of phagocytosing and destroying the parasite,^75^ initiating processes such as phagolysosome formation, release of degradative enzymes, oxidative burst generation, and nitric oxide production.^76^ TEM images supported these findings, showing high metabolic activity in macrophages and dense vesicular deposition within parasitophorous vacuoles.

*Leishmania* spp. are highly adapted for survival and replication in both the sandfly vector and the macrophage host, deploying distinct gene and protein repertoires to thrive in each environment. While many mRNAs are constitutively expressed, virulence and pathogenesis may depend on nutrient availability,^77,78^ which could explain differences in **BT-Ac** susceptibility between promastigotes and amastigotes. Given methodological limitations in evaluating intracellular amastigotes, the exact intrinsic death pathway in these forms remains to be elucidated.

Volume electron microscopy allowed a high-resolution visualization of alterations in the promastigote cell body. This technique is a powerful tool for exploring the organization of structures that are otherwise difficult to access, such as the unique organelles of microorganisms.^79,80^ In trypanosomatids, 3D reconstruction has emerged as an important approach to study key structures, including the cytostome-cytopharynx,^81^ the contractile vacuole,^82,83^ and the flagellum.^84^ With this same technique, we have showed that **BT-Ac** caused unprecedented alterations in the organization of microtubules and membrane association, causing repetitive patterns of irregularities. These structural alterations have not been documented in literature so far and interestingly, this same drug **BT-Ac** has not caused this in *T. cruzi*.^22^

Some data supports that microtubules are conserved across trypanosomatids^85^ but microtubule associated proteins (MAP) are poorly investigated. MAPs are proteins responsible to stabilize and bundle microtubules into persistent structures but are often associated with intermicrotubule cross-linking.^86^ Most of the MAPs investigated to date are from *T. brucei* and little is known about how they function to build, maintain, and remodel the array. One MAP identified in *T. brucei* was associated with the attachment of the microtubules with the plasma membrane. The MAP WCB determines the stable connection of the subpellicular corset and its depletion disrupts the spatial organization across the cell surface.^87^

Taken together, our results support a model in which **BT-Ac** exposure induces early ROS/RNS overproduction, leading to oxidative damage of lipids, proteins, and organelles. This initiates lipid peroxidation, ER stress, and mitochondrial dysfunction, destabilizing cytoskeletal and membrane structures, and disrupting homeostatic organelles such as acidocalcisomes. These events culminate in parasite death via apoptosis-like and/or autophagic pathways in promastigotes, while in amastigotes, death occurs predominantly through macrophage-mediated effector mechanisms. The novel ultrastructural alterations reported here warrant further molecular investigations to clarify the detailed mechanism of **BT-Ac** action in *L. amazonensis*. These results highlight the importance of parallel efforts: advancing drug discovery while simultaneously deepening the understanding of parasite cell biology.

## 5 Conclusion

In conclusion, we demonstrated that **BT-Ac** has selective antileishmanial activity and that treatment actively induced functional loss in *L. amazonensis* parasites, with the main mechanism of action involving the induction of oxidative stress, triggering apoptosis-like and/or autophagic death in promastigotes. In contrast, amastigote death was a consequence of the effector activity of macrophages, which were activated by **BT-Ac**. However, no reports in the literature were found regarding the novel ultrastructural changes observed in this study; therefore, further molecular-level investigations are required to establish the mechanism of action of **BT-Ac** on *L. amazonensis* parasitic forms.
